# Marine-Derived Neoagarotetraose Alleviates Dry Eye Disease by Suppressing Inflammation and Apoptosis in a Murine Model

**DOI:** 10.3390/md24050175

**Published:** 2026-05-12

**Authors:** Nan Wu, Yating Du, Chaocheng Wu, Zhuhua Chan, Runying Zeng

**Affiliations:** 1 Technology Innovation Center for Exploitation of Marine Biological Resources, Third Institute of Oceanography, Ministry of Natural Resources, Xiamen 361005, China; wunan@tio.org.cn (N.W.); duyating@tio.org.cn (Y.D.); wuchaocheng@tio.org.cn (C.W.); 2 Fujian Provincial Key Laboratory of Island Conservation and Development, Island Research Center, Ministry of Natural Resources, Pingtan 350400, China

**Keywords:** neoagarotetraose, dry eye disease, ocular surface inflammation, anti-apoptosis, corneal epithelial protection

## Abstract

Dry eye disease (DED) is a complex ocular surface disorder characterized by tear film instability, chronic inflammation, and epithelial damage, for which current treatments remain limited. Marine-derived bioactive oligosaccharides have attracted increasing interest due to their diverse pharmacological activities and favorable safety profiles. In this study, we investigated the therapeutic potential of neoagarotetraose (NA4), a marine oligosaccharide derived from red algal agar, in a murine model of DED. DED was induced in eight-week-old female C57BL/6 mice by topical instillation of 0.2% benzalkonium chloride for seven consecutive days. NA4 was administered topically at concentrations of 125, 250, and 500 mg/L. Therapeutic outcomes were evaluated by tear secretion, corneal fluorescein staining, histopathological analysis, immunofluorescence staining for Ki67, F4/80, IL-1β, IL-6, and TNF-α, TUNEL assay for apoptosis, and ELISA for cytokine levels. NA4 treatment significantly improved tear secretion and reduced corneal fluorescein staining scores. Histological analysis revealed that NA4 preserved corneal epithelial thickness and restored conjunctival goblet cell density. Immunofluorescence analysis revealed that NA4 reversed inflammation-associated epithelial hyperproliferation and attenuated macrophage infiltration. Moreover, NA4 markedly suppressed the expression and tissue levels of IL-1β, IL-6, and TNF-α, and attenuated corneal epithelial apoptosis, with the 500 mg/L NA4 group showing no significant difference in efficacy compared to the positive control 0.1% sodium hyaluronate. These findings demonstrate that NA4, a marine-derived oligosaccharide, exerts multi-targeted protective effects against DED by improving tear film stability, preserving ocular surface integrity, suppressing inflammation, and reducing apoptosis. Our study highlights the potential of marine oligosaccharides such as NA4 as promising candidates for ocular surface disease management and supports the further exploration of marine resources for ophthalmic therapeutic applications.

## 1. Introduction

Dry eye disease (DED) is a multifactorial disorder of the ocular surface characterized by tear film instability, hyperosmolarity, ocular surface inflammation and damage, accompanied by symptoms such as dryness, burning sensation, and visual disturbance. Epidemiological studies estimate that DED affects approximately 5% to 50% of the global population, significantly impairing patients’ quality of life and imposing a substantial socioeconomic burden [1,2]. The pathophysiology of DED involves a complex interplay between inflammation, oxidative stress, and apoptosis, creating a self-perpetuating cycle that exacerbates ocular surface damage. Disruption of tear film homeostasis leads to epithelial apoptosis, loss of goblet cells, and activation of inflammatory cascades, ultimately compromising the integrity and function of the ocular surface [3,4,5].

Current treatment strategies for DED primarily focus on tear supplementation, anti-inflammatory therapy, and immunomodulation. Artificial tears provide temporary symptomatic relief but often contain preservatives that may aggravate corneal irritation. Cyclosporine A, an immunomodulatory agent, has been approved for DED treatment, yet its efficacy varies among patients and its onset of action is delayed. Corticosteroids offer potent anti-inflammatory effects but are associated with significant adverse effects, including elevated intraocular pressure and increased susceptibility to infection [6,7,8,9,10]. Given these limitations, there is an urgent need to explore novel therapeutic agents with improved safety profiles and multi-targeted mechanisms for the effective management of DED.

Marine-derived bioactive compounds have garnered increasing attention due to their diverse pharmacological activities, favorable biocompatibility, and structural uniqueness [11,12]. Neoagarotetraose (NA4), a low-molecular-weight oligosaccharide derived from the enzymatic hydrolysis of agar, is a natural product extracted from marine red algae. As a marine oligosaccharide with a well-defined structure, NA4 possesses excellent water solubility and low toxicity, making it an attractive candidate for biomedical applications. Previous studies have demonstrated that NA4 exhibits potent antioxidant and anti-inflammatory properties, effectively scavenging reactive oxygen species, reducing oxidative stress, and suppressing the production of pro-inflammatory cytokines in various experimental models [13,14,15]. Additionally, NA4 has been shown to promote cell proliferation and tissue repair, suggesting its potential for use in treating inflammatory and degenerative diseases [16]. Despite these well-documented bioactivities, the therapeutic potential of NA4 in ocular surface disorders, particularly DED, remains largely unexplored.

In this study, we evaluated the therapeutic effects of NA4 in a murine DED model, focusing on tear secretion, corneal fluorescein staining, histopathological changes, and the expression of pro-inflammatory cytokines (IL-1β, IL-6, TNF-α) and apoptotic cells. Our results showed that NA4 treatment improved tear secretion, reduced corneal damage, restored goblet cell density, and attenuated abnormal epithelial proliferation. Mechanistically, NA4 suppressed macrophage infiltration, reduced inflammatory cytokine expression, and attenuated corneal apoptosis. These findings suggest that NA4, as a marine-derived oligosaccharide, exerts multi-targeted protective effects against DED, highlighting its potential as a novel therapeutic candidate derived from marine resources for ocular surface disease management.

## 2. Results

### 2.1. NA4 Restores Tear Secretion and Corneal Integrity in DED Mice

**Figure 1:**
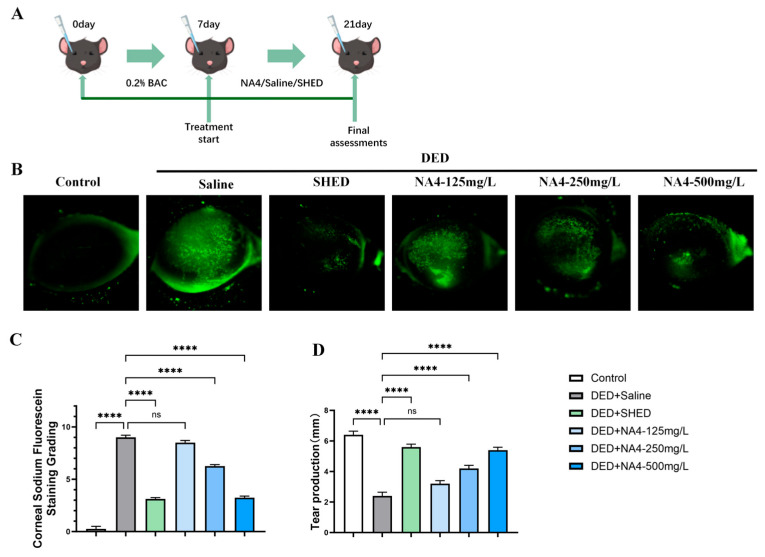
Therapeutic effects of NA4 on tear secretion and corneal integrity in DED mice. (**A**) Schematic illustration of the experimental design. (**B**) Representative corneal fluorescein staining images. (**C**) Quantitative analysis of corneal fluorescein staining scores. (**D**) Tear production measured by phenol red thread test. Data are presented as mean ± SEM (*n* = 6). For (**C**), Kruskal–Wallis test with Dunn’s post hoc test was used; for (**D**), one-way ANOVA with Tukey’s post hoc test was used. Significance brackets compare each treatment group to the DED + Saline group (unless otherwise indicated by a horizontal line). **** *p* < 0.0001; ns, not significant.

The therapeutic efficacy of NA4, a marine-derived oligosaccharide obtained from red algal agar, was evaluated in a BAC-induced mouse model of DED. As shown in Figure 1D, tear secretion was significantly reduced in the DED + Saline group compared with the Control group (*p* < 0.0001). Treatment with NA4 increased tear secretion. The NA4-125 mg/L group showed a marginal increase compared with the DED + Saline group (*p* = 0.0532), whereas the NA4-250 mg/L and NA4-500 mg/L groups achieved significant improvements (both *p* < 0.0001), with the 500 mg/L group showing no statistically significant difference from the DED + SHED group.

Corneal fluorescein staining revealed extensive epithelial defects in the DED + Saline group compared with the Control group (*p* < 0.0001). NA4 treatment at 250 mg/L and 500 mg/L significantly reduced staining scores compared with the DED + Saline group (both *p* < 0.0001), whereas the 125 mg/L dose did not produce a significant effect (*p* = 0.2366; Figure 1B,C). Collectively, these results demonstrate that NA4 effectively alleviates tear deficiency and restores corneal epithelial integrity in DED mice, and that this effect is pharmacological rather than attributable to the vehicle.

### 2.2. NA4 Ameliorates Corneal and Conjunctival Histopathological Changes

**Figure 2:**
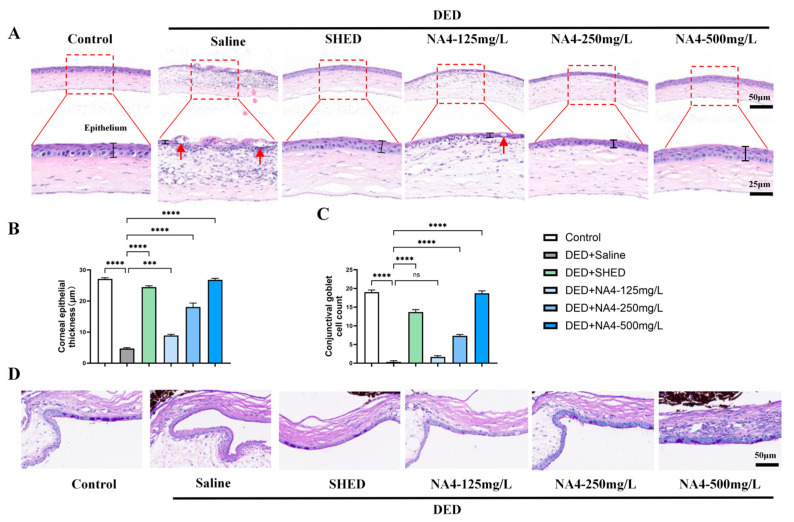
Protective effects of NA4 on corneal and conjunctival histopathology in DED mice. (**A**) Representative H&E staining of corneal sections. Red arrows indicate epithelial thinning and disorganization. Scale bars: 50 μm (**top**), 25 μm (**bottom**). (**B**) Quantitative analysis of corneal epithelial thickness. (**C**) Representative PAS staining of conjunctival sections showing goblet cell morphology and distribution. (**D**) Quantitative analysis of conjunctival goblet cell density. Data are presented as mean ± SEM (*n* = 6). One-way ANOVA with Tukey’s post hoc test was used for (**B**,**D**). Significance brackets compare each treatment group to the DED + Saline group unless otherwise indicated. *** *p* < 0.001; **** *p* < 0.0001; ns, not significant.

The protective effects of NA4, a marine-derived oligosaccharide, on ocular surface structures were evaluated by histological analysis. H&E staining revealed significant corneal epithelial thinning in the DED + Saline group compared with the Control group (*p* < 0.0001; Figure 2A,B). NA4 treatment preserved corneal epithelial thickness. The 125 mg/L group showed significant improvement (*p* = 0.0008 vs. DED + Saline) and the 250 mg/L and 500 mg/L groups achieved more pronounced restoration (both *p* < 0.0001), with the 500 mg/L group reaching levels similar to the Control. PAS staining demonstrated a marked reduction in conjunctival goblet cell density in the DED + Saline group (*p* < 0.0001 vs. Control; Figure 2C,D). The NA4-125 mg/L group did not significantly restore goblet cell counts (*p* = 0.2824 vs. DED + Saline), whereas the 250 mg/L and 500 mg/L groups achieved significant improvements (both *p* < 0.0001). Notably, the NA4-500 mg/L group differed significantly from SHED (*p* = 0.0283), restoring goblet cell counts to near-normal levels. These findings indicate that NA4, as a marine-derived oligosaccharide, protects against corneal epithelial atrophy and conjunctival goblet cell loss in DED.

### 2.3. NA4 Suppresses Ocular Surface Inflammation and Restores Epithelial Proliferation

**Figure 3:**
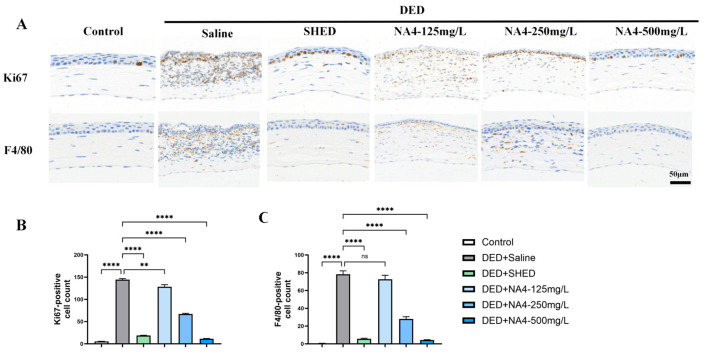
Effects of NA4 on inflammation and epithelial proliferation in DED mouse corneas. (**A**) Representative immunohistochemical staining for Ki67 (**upper**) and F4/80 (**lower**) in corneal sections. Scale bar: 50 μm. (**B**) Quantification of Ki67-positive cells per field. (**C**) Quantification of F4/80-positive cells per field. Data are presented as mean ± SEM (*n* = 6). One-way ANOVA with Tukey’s post hoc test was used for (**B**,**C**). Significance brackets compare each treatment group to the DED + Saline group. ** *p* < 0.01; **** *p* < 0.0001; ns, not significant.

Immunohistochemical staining for Ki67 (cell proliferation) and F4/80 (macrophage infiltration) was performed to evaluate the anti-inflammatory and tissue-modulating effects of NA4, a marine-derived oligosaccharide. As shown in Figure 3, the DED + Saline group exhibited significant increases in both Ki67-positive and F4/80-positive cells compared with the Control group (both *p* < 0.0001), indicating inflammation-associated epithelial hyperproliferation and macrophage infiltration. For Ki67, NA4 treatment reduced Ki67-positive cells. The 125 mg/L group achieved a significant reduction (*p* = 0.0012 vs. DED + Saline), and the 250 mg/L and 500 mg/L groups also showed significant reductions (both *p* < 0.0001 vs. DED + Saline). For F4/80, the NA4-125 mg/L group did not significantly reduce macrophage infiltration (*p* = 0.4420 vs. DED + Saline), whereas the 250 mg/L and 500 mg/L groups achieved significant reductions (both *p* < 0.0001 vs. DED + Saline). These results demonstrate that NA4 effectively alleviates inflammatory cell infiltration and restores normal epithelial homeostasis in DED.

### 2.4. NA4 Attenuates Apoptosis in the Corneal Epithelium

**Figure 4:**
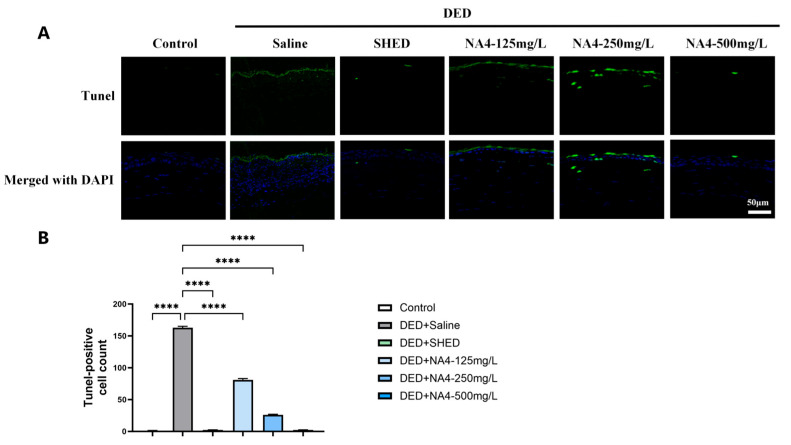
Anti-apoptotic effects of NA4 on corneal epithelium in DED mice. (**A**) Representative TUNEL staining (green) and merged images with DAPI (blue) showing apoptotic cells in corneal sections. Scale bar: 50 μm. (**B**) Quantification of TUNEL-positive cells per field. Data are presented as mean ± SEM (*n* = 6). One-way ANOVA with Tukey’s post hoc test was used for (**B**). Significance brackets compare each treatment group to the DED + Saline group. **** *p* < 0.0001.

Apoptosis plays a critical role in DED pathogenesis. TUNEL staining revealed a significant increase in apoptotic cells in the DED + Saline group compared with the Control group (*p* < 0.0001; Figure 4). Treatment with NA4 reduced TUNEL-positive cells, with all concentrations achieving significant reductions compared with the DED + Saline group (125 mg/L, *p* < 0.0001; 250 mg/L, *p* < 0.0001; 500 mg/L, *p* < 0.0001). The 500 mg/L group restored apoptosis levels to near-baseline and showed a statistically significant difference compared to the SHED group (*p* = 0.0176). These findings indicate that NA4 exerts potent anti-apoptotic effects in the corneal epithelium of DED mice.

### 2.5. NA4 Reduces Proinflammatory Cytokine Expression in the Ocular Surface

**Figure 5:**
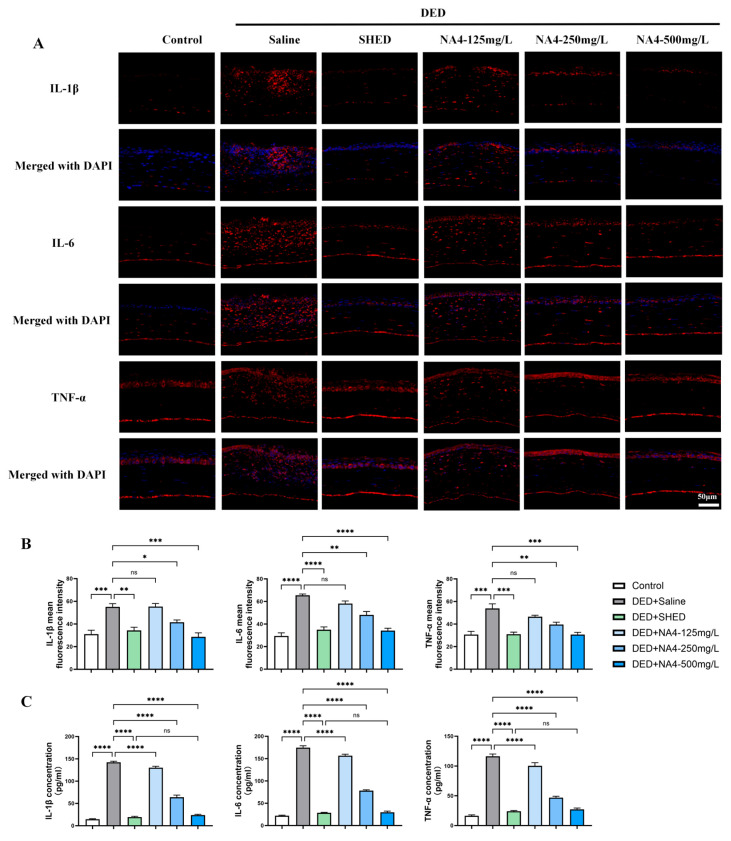
NA4 suppresses proinflammatory cytokine expression in DED mouse corneas. (**A**) Representative immunofluorescence images showing IL-1β, IL-6, and TNF-α expression (red) with DAPI nuclear counterstaining (blue). Scale bar: 50 μm. (**B**) Quantitative analysis of mean fluorescence intensity for IL-1β, IL-6, and TNF-α. (**C**) ELISA quantification of IL-1β, IL-6, and TNF-α protein concentrations in corneal tissue homogenates. Data are presented as mean ± SEM (*n* = 6). For (**B**,**C**), one-way ANOVA with Tukey’s post hoc test was used. Significance brackets compare each treatment group to the DED + Saline group unless otherwise indicated (e.g., bracket between Control and DED compares these two groups directly). * *p* < 0.05; ** *p* < 0.01; *** *p* < 0.001; **** *p* < 0.0001; ns, not significant.

The expression of IL-1β, IL-6, and TNF-α in corneal tissues was assessed to further evaluate the anti-inflammatory effects of NA4, a marine-derived oligosaccharide. Immunofluorescence staining revealed elevated levels of IL-1β, IL-6, and TNF-α in the DED + Saline group compared with the Control group. For IL-1β, the DED + Saline group showed significant elevation (*p* = 0.0004 vs. Control), and NA4 treatment significantly reduced fluorescence intensity at 250 mg/L (*p* = 0.0260) and 500 mg/L (*p* = 0.0002), whereas the 125 mg/L group showed no significant effect (*p* > 0.9999). For IL-6, the DED + Saline group showed significant elevation (*p* < 0.0001 vs. Control), and NA4 treatment significantly reduced fluorescence intensity at 250 mg/L (*p* = 0.0010) and 500 mg/L (*p* < 0.0001), whereas the 125 mg/L group showed no significant effect (*p* = 0.1702). For TNF-α, the DED + Saline group showed significant elevation (*p* = 0.0001 vs. Control), and NA4 treatment significantly reduced fluorescence intensity at 250 mg/L (*p* = 0.0073) and 500 mg/L (*p* = 0.0001), whereas the 125 mg/L group showed no significant effect (*p* = 0.2076; Figure 5A,B).

Consistently, ELISA analysis of corneal tissue homogenates demonstrated that NA4 reduced elevated cytokine levels. For all three cytokines, the NA4-500 mg/L group significantly decreased IL-1β, IL-6, and TNF-α concentrations compared with the DED + Saline group (all *p* < 0.0001). Notably, no significant differences were observed between the NA4-500 mg/L and SHED groups for IL-1β (*p* = 0.1441), IL-6 (*p* = 0.9789), or TNF-α (*p* = 0.5763). These data indicate that NA4-500 mg/L and SHED did not differ significantly in reducing these cytokines under the current experimental conditions (Figure 5C). Collectively, these results demonstrate that NA4 effectively suppresses local inflammation in DED.

## 3. Discussion

DED is a prevalent ocular surface disorder characterized by tear film instability, inflammation, and epithelial damage, which significantly impairs patients’ quality of life. Current therapeutic options, including artificial tears, cyclosporine A, and corticosteroids, are often limited by delayed onset, variable efficacy, and adverse effects such as ocular irritation and poor bioavailability [17]. In recent years, marine-derived bioactive compounds have attracted considerable attention as potential therapeutic agents for inflammatory diseases. For instance, fucoidan, a sulfated polysaccharide extracted from brown algae, has demonstrated potent anti-inflammatory activities through suppression of neutrophil migration and pro-inflammatory mediator production in experimental models of inflammation [18,19]. Similarly, porphyran, a polysaccharide derived from the red alga Porphyra yezoensis, has demonstrated antioxidant and anti-inflammatory activities that may confer protective effects against inflammatory tissue damage [20]. These findings highlight the potential of marine-derived oligosaccharides and polysaccharides as promising candidates for DED treatment due to their favorable safety profiles, structural diversity, and multi-targeted bioactivities. In this study, we demonstrated for the first time that NA4, a marine-derived oligosaccharide obtained from red algal agar, exerts therapeutic effects in a murine model of DED by improving tear secretion, preserving corneal and conjunctival integrity, suppressing inflammation, and reducing apoptosis.

Our findings revealed that NA4 treatment significantly improved tear secretion and prolonged tear film stability in BAC-induced DED mice. Compared with other marine-derived oligosaccharides and polysaccharides that have shown promise for ocular surface therapy, NA4 exhibits distinct structural and functional characteristics that may confer therapeutic advantages. Fucoidan, a high-molecular-weight sulfated polysaccharide (typically 50–3700 kDa) derived from brown algae, has demonstrated anti-apoptotic and anti-inflammatory effects in dry eye models by suppressing hyperosmotic stress-induced corneal epithelial cell death and reducing tear hyposecretion when administered orally [21]. However, the high molecular weight and viscosity of fucoidan may limit its corneal penetration and bioavailability when applied topically [22]. Similarly, chitosan oligosaccharides (COS), typically with degrees of polymerization (DP) ranging from 2 to 7, have exhibited mucoadhesive properties and anti-inflammatory activity, with COS6 (DP 6) identified as the most effective anti-inflammatory form among tested chitooligosaccharides [23]. Notably, in comparative studies of agaro-oligosaccharides with varying DPs (2–10), NA4 demonstrated the strongest anti-inflammatory capacity among the tested analogs, most effectively suppressing LPS-induced IL-1β, TNF-α, IL-6, and iNOS expression [24]. In our study, NA4 exhibited potent efficacy at concentrations as low as 125–500 mg/L, which is considerably lower than the effective concentrations typically required for high-molecular-weight fucoidan [21]. This superior potency may be attributed to the optimal DP 4 structure of NA4, which balances water solubility and membrane permeability, enabling efficient penetration through the corneal epithelium to exert multi-targeted protective effects. Furthermore, unlike chitosan derivatives that primarily function through mucoadhesive glycocalyx formation [25], NA4 appears to act through direct cellular signaling mechanisms involving proliferation promotion, apoptosis inhibition, and cytokine suppression, suggesting a distinct mechanism of action among marine-derived ocular therapeutics. Thus, among marine-derived oligosaccharides, the tetrameric structure of NA4 (DP4) offers an optimal balance of physicochemical and biological properties for ocular surface therapy.

Histopathological analysis further corroborated the protective effects of NA4 on the ocular surface. The significant restoration of conjunctival goblet cell density following NA4 treatment is particularly noteworthy, as goblet cell loss is a hallmark of DED pathogenesis and directly contributes to tear film instability [26]. Similar to findings reported for hyaluronic acid-based therapies in the context of DED [27], NA4 treatment reversed goblet cell depletion, suggesting that NA4 may regulate mucin production and secretion. The parallel improvement in both corneal epithelial thickness and goblet cell density implies that NA4 exerts broad-spectrum protective effects on ocular surface tissues, potentially through mechanisms involving the regulation of cell proliferation and differentiation [1].

Inflammation is widely recognized as a central driver of the DED vicious cycle [28]. Our study demonstrated that NA4 treatment significantly reduced the infiltration of F4/80-positive macrophages [29] and suppressed the expression of pro-inflammatory cytokines IL-1β, IL-6, and TNF-α in corneal tissues. These anti-inflammatory effects are consistent with previous studies reporting that NA4 and related marine oligosaccharides inhibit the production of inflammatory mediators in macrophages and other cell types [14]. Notably, the efficacy of NA4-500 mg/L in reducing cytokine levels did not differ significantly from that of the positive control, highlighting its potential as an effective anti-inflammatory agent for DED.

Apoptosis of corneal and conjunctival epithelial cells plays a critical role in the pathogenesis of DED, contributing to barrier disruption and persistent inflammation [28]. Our TUNEL staining results revealed a marked increase in apoptotic cells in the corneas of DED mice [30], which was significantly reduced by NA4 treatment. The concurrent reduction in inflammatory cytokines further supports the notion that NA4 disrupts the inflammatory-apoptotic feedback loop that perpetuates DED progression [31]. Compared with the effects of other natural product-based interventions reported in the literature, such as polyphenols, flavonoids, and bioactive peptides that mitigate ocular surface damage through anti-apoptotic mechanisms [30], NA4 demonstrated comparable efficacy in reducing apoptosis, suggesting that NA4 may represent a novel therapeutic option with a multi-targeted mechanism of action.

The mechanisms underlying the therapeutic effects of NA4 in DED are likely multi-faceted. In the DED model, Ki67 expression was abnormally elevated, reflecting inflammation-associated hyperproliferation of the corneal epithelium. NA4 treatment reduced Ki67-positive cells back to baseline levels, suggesting that NA4 suppresses this pathological hyperproliferation and restores normal epithelial homeostasis. Ki67 is an established marker of cell proliferation [32], and its reduction toward control levels in the NA4-treated groups indicates a normalization of epithelial turnover under inflammatory conditions. These findings suggest that NA4 exerts integrated protective effects on the ocular surface by simultaneously targeting inflammation, oxidative stress, and pathological epithelial hyperproliferation, a multi-targeted approach that aligns with the complex pathophysiology of DED and reflects the emerging paradigm of mechanism-based combination therapies [33].

Several limitations of this study should be acknowledged. First, the precise molecular mechanisms by which NA4 exerts its anti-inflammatory and anti-apoptotic effects remain to be fully elucidated. Future studies using pathway-specific inhibitors or genetic approaches are warranted. Second, the BAC-induced DED model represents a toxic epitheliopathy and does not capture the full spectrum of human DED (e.g., meibomian gland dysfunction, neural abnormalities, autoimmune components). Our findings are therefore most directly applicable to DED subtypes driven by epithelial injury and inflammation. Third, only female C57BL/6 mice were used in this study. Female mice exhibit more severe DED phenotypes (greater corneal staining, inflammation, and apoptosis) than males in multiple models, supporting their utility as the more sensitive model for efficacy testing [34]. Nevertheless, this choice limits generalizability to male subjects; future studies should include both sexes to confirm NA4’s therapeutic effects across sexes. Fourth, NA4 was administered topically; further optimization of formulation and delivery route may enhance its clinical applicability. Fifth, although NA4 showed promising efficacy in a murine model, its safety and efficacy in human DED patients require validation in well-designed clinical trials.

## 4. Materials and Methods

### 4.1. Animals and Grouping

Eight-week-old female C57BL/6 mice (weighing 18–22 g) were purchased from Xiamen Fudexin (Xiamen, China). All animal experiments were approved by the Institutional Animal Care and Use Committee of the Third Institute of Oceanography, Ministry of Natural Resources (Approval No. TIO-IACUC-03-2025-07-09) and complied with the ARVO Statement for the Use of Animals in Ophthalmic and Vision Research. Mice were housed under standard laboratory conditions with a 12-h light/dark cycle and free access to food and water. The sample size of 6 mice per group was chosen based on previous published murine models of dry eye disease [35] and is consistent with institutional guidelines and standard practice in ocular surface research, which typically uses 5–8 animals per group for histological and biochemical analyses. Only the right eye of each mouse was used for DED induction and all subsequent assessments; the left eye served as an untreated internal control and was not included in the data analysis. Mice were randomly assigned to experimental groups using a computer-generated random number sequence. All outcome assessments, including fluorescein staining scoring, cell counting (Ki67, F4/80, TUNEL, and goblet cells), and fluorescence intensity quantification, were performed by an investigator blinded to the group allocation. The blinding was maintained until all data were analyzed.

Humane endpoints, analgesia, and euthanasia: Mice were monitored daily. Predefined humane endpoints (>20% weight loss, persistent lethargy, inability to reach food/water, or severe corneal opacity/ulceration) were not reached. No analgesia was administered because BAC-induced DED causes only mild, transient ocular surface irritation without sustained pain. This is consistent with previous BAC-based dry eye studies [35,36]. At experiment end, mice were deeply anesthetized with isoflurane (5% induction, 2% maintenance; RWD Life Science Co., Ltd., Shenzhen, China) and euthanized by cervical dislocation, followed by death confirmation via cardiac arrest.

### 4.2. NA4 Preparation and Administration

NA4 was prepared by enzymatic hydrolysis of agarose using a recombinant β-agarase as previously described [37,38]. The hydrolysate was decolorized with activated carbon, desalted with a cation-exchange resin, and fractionated by Bio-Gel P2 fine gel filtration chromatography (Bio-Rad Laboratories, Inc., Hercules, CA, USA). The purified NA4 showed a single peak at a retention time consistent with the NA4 standard (14.304 min), and its purity was determined to be 96.53% by peak area normalization. Electrospray ionization time-of-flight mass spectrometry (ESI-TOF-MS, Waters Corporation, Milford, MA, USA) confirmed the degree of polymerization as 4, with [M+Na]^+^ and [M+H]^+^ peaks at m/z 653.1862 and 629.1918, respectively, matching the theoretical molecular weight of NA4 (630.55 Da). Endotoxin levels measured by LAL assay were below 0.1 EU/mL. For eye-drop formulations, NA4 was freshly dissolved in sterile saline to achieve 125, 250, and 500 mg/L; pH ranged from 6.9 to 7.1, and osmolality from 295 to 315 mOsm/kg. Mice in the treatment groups received these NA4 eye drops (5 μL per eye) three times daily for 14 consecutive days. The selected NA4 concentrations (125–500 mg/L) were based on preliminary dose–response tests and prior literature [14], covering sub-therapeutic to fully therapeutic ranges.

### 4.3. Induction of Dry Eye Disease and Experimental Groups

DED was induced by topical instillation of 0.2% benzalkonium chloride (BAC) solution (5 μL per eye, three times daily) only into the right eye for seven consecutive days. The left eye received no treatment and served as an untreated internal control; it was not used for any experimental measurements. Mice were randomly divided into six groups (*n* = 6 per group): Control (no treatment), DED + saline (sterile saline eye drops, vehicle control), positive control (preservative-free 0.1% sodium hyaluronate eye drops, SHED), and NA4 treatment groups (125, 250, and 500 mg/L). All treatments were administered topically three times daily for 14 days following BAC induction.

### 4.4. Tear Secretion Measurement

Tear secretion was assessed using the phenol red cotton thread method. In unanesthetized mice, a phenol red thread (Tianjin Jingming Company, Tianjin, China) was placed into the conjunctival sac for 30 s. The length of the red-stained portion was measured under a microscope (Nikon Corporation, Tokyo, Japan). Measurements were performed three times, and the average value was recorded.

### 4.5. Corneal Fluorescein Staining and Scoring

Mice were anesthetized by intraperitoneal injection of 1% pentobarbital sodium. A 1% fluorescein sodium solution was instilled into the conjunctival sac, and the mice were allowed to blink to ensure even distribution. After 1 min, the cornea was examined under a slit-lamp microscope (Haag-Streit Diagnostics, Wedel, Germany) with cobalt blue light. Corneal staining was scored by dividing the cornea into four quadrants. The scoring criteria were as follows: 0, no staining; 1, mild punctate staining; 2, moderate punctate staining; 3, dense punctate staining; and 4, confluent staining or plaque formation. The total score was calculated as the sum of the four quadrants.

### 4.6. Histological Staining (H&E and PAS)

After euthanasia, eyeballs were enucleated and fixed in 4% paraformaldehyde for 24 h, followed by dehydration, paraffin embedding, and sectioning. For hematoxylin and eosin (H&E) staining, sections were deparaffinized, stained with hematoxylin and eosin (Solarbio, Beijing, China), and observed under a light microscope (Nikon Inc., Melville, NY, USA). Corneal epithelial thickness was measured using ImageJ software (version 1.54, National Institutes of Health, Bethesda, MD, USA).

For periodic acid–Schiff (PAS) staining, sections were deparaffinized, oxidized with periodic acid, stained with Schiff’s reagent (Solarbio), and counterstained with hematoxylin. Goblet cells in the conjunctival epithelium were counted in five randomly selected non-overlapping fields per section under a light microscope at ×400 magnification (scale bar = 50 μm). Counting was performed by an examiner blinded to the experimental groups.

### 4.7. Immunofluorescence Staining

Sections were deparaffinized, rehydrated, subjected to antigen retrieval in citrate buffer, and blocked with 5% bovine serum albumin, followed by overnight incubation at 4 °C with primary antibodies against Ki67, F4/80, IL-1β, IL-6, and TNF-α (all 1:200, Aifang Biotechnology, Changsha, China). After washing, sections were incubated with fluorescent secondary antibodies (1:500, Servicebio, Wuhan, China) for 1 h at room temperature, counterstained with DAPI, and imaged using an inverted fluorescence microscope (ECLIPSE Ti2, Nikon Corporation, Tokyo, Japan). Fluorescence intensity was quantified using ImageJ software.

### 4.8. TUNEL Staining

Apoptotic cells in corneal sections were detected using a TUNEL assay kit (Roche, Basel, Switzerland) according to the manufacturer’s instructions. Briefly, sections were deparaffinized, rehydrated, and treated with proteinase K (Beyotime, Shanghai, China). After washing, sections were incubated with the TUNEL reaction mixture for 60 min at 37 °C in the dark. Nuclei were counterstained with DAPI. TUNEL-positive cells were counted in five randomly selected fields per section under a fluorescence microscope.

### 4.9. Enzyme-Linked Immunosorbent Assay (ELISA)

Corneal tissues were homogenized in RIPA lysis buffer (Beyotime) and centrifuged at 12,000 rpm for 10 min. The supernatants were collected for cytokine analysis. Levels of IL-1β, IL-6, and TNF-α were measured using commercial ELISA kits (Aifang Biotechnology, Changsha, China) according to the manufacturer’s protocols. Absorbance was read at 450 nm using a microplate reader (Agilent BioTek, Santa Clara, CA, USA).

### 4.10. Statistical Analysis

All data are presented as mean ± SEM. Statistical analyses were performed using GraphPad Prism 9.0. For normally distributed continuous data (tear secretion, corneal epithelial thickness, goblet cell density, cell counts, fluorescence intensity, ELISA), comparisons among multiple groups were conducted using one-way ANOVA followed by Tukey’s multiple comparisons test. For ordinal data (corneal fluorescein staining scores), the Kruskal–Wallis test followed by Dunn’s multiple comparisons test was used. A *p* value < 0.05 was considered statistically significant.

## 5. Conclusions

In conclusion, this study provides the first evidence that NA4, a marine-derived oligosaccharide, exerts therapeutic effects in a murine model of DED by improving tear film stability, preserving corneal and conjunctival integrity, suppressing inflammation, and reducing apoptosis. These multi-targeted protective effects position NA4 as a promising candidate for DED treatment. With further mechanistic investigation and formulation development, NA4 may emerge as a novel, safe, and effective therapeutic option derived from marine resources for the management of DED.

## Data Availability

The data that support the findings of this study are available from the corresponding author upon reasonable request.

## Abbreviations

The following abbreviations are used in this manuscript:

DED	Dry eye disease
NA4	Neoagarotetraose
BAC	Benzalkonium chloride
H&E	Hematoxylin and eosin
PAS	Periodic acid–Schiff
TUNEL	Terminal deoxynucleotidyl transferase dUTP nick end labeling
ELISA	Enzyme-linked immunosorbent assay
SHED	Sodium hyaluronate eye drops
SEM	Standard error of the mean
ns	Not significant
ROS	Reactive oxygen species
